# Genomic landscapes of canine splenic angiosarcoma (hemangiosarcoma) contain extensive heterogeneity within and between patients

**DOI:** 10.1371/journal.pone.0264986

**Published:** 2022-07-22

**Authors:** Shukmei Wong, E. J. Ehrhart, Samuel Stewart, Victoria Zismann, Jacob Cawley, Rebecca Halperin, Natalia Briones, Keith Richter, Karthigayini Sivaprakasam, Nieves Perdigones, Tania Contente-Cuomo, Salvatore Facista, Jeffrey M. Trent, Muhammed Murtaza, Chand Khanna, William P. D. Hendricks

**Affiliations:** 1 Translational Genomics Research Institute, Phoenix, Arizona, United States of America; 2 Charles River Laboratories, Wilmington, MA, United States of America; 3 Ethos Discovery, San Diego, CA, United States of America; 4 Ethos Veterinary Health, Woburn, MA, United States of America; 5 Department of Genetics and Genome Sciences, Case Western Reserve University, Cleveland, Ohio, United States of America; Colorado State University, UNITED STATES

## Abstract

Cancer genomic heterogeneity presents significant challenges for understanding oncogenic processes and for cancer’s clinical management. Variation in driver mutation frequency between patients with the same tumor type as well as within an individual patients’ cancer can shape the use of mutations as diagnostic, prognostic, and predictive biomarkers. We have characterized genomic heterogeneity between and within canine splenic hemangiosarcoma (HSA), a common naturally occurring cancer in pet dogs that is similar to human angiosarcoma (AS). HSA is a clinically, physiologically, and genomically complex canine cancer that may serve as a valuable model for understanding the origin and clinical impact of cancer heterogeneity. We conducted a prospective collection of 52 splenic masses from 43 dogs (27 HSA, 15 benign masses, and 1 stromal sarcoma) presenting for emergency care with hemoperitoneum secondary to a ruptured splenic mass. Multi-platform genomic analysis included matched tumor/normal targeted sequencing panel and exome sequencing. We found candidate somatic cancer driver mutations in 14/27 (52%) HSAs. Among recurrent candidate driver mutations, *TP53* was most commonly mutated (30%) followed by *PIK3CA* (15%), *AKT1* (11%), and *CDKN2AIP* (11%). We also identified significant intratumoral genomic heterogeneity, consistent with a branched evolution model, through multi-region exome sequencing of three distinct tumor regions from selected primary splenic tumors. These data provide new perspectives on the genomic landscape of this veterinary cancer and suggest a cross-species value for using HSA in pet dogs as a naturally occurring model of intratumoral heterogeneity.

## Introduction

Dramatic variation in clinical, cellular, and genomic cancer features is common across patients with the same tumor type as well as within tumors in individual patients. Such heterogeneity presents challenges for uniform diagnosis, prognosis, and treatment planning for many cancers [1]. Intratumoral heterogeneity (ITH) arising from branched evolution holds particular relevance for molecular and genomic diagnostics because it can lead to spatial variation in the abundance of mutations that may serve as biomarkers. Thus, especially in tumors that also bear cellular heterogeneity (e.g. with abundant stromal or vascular components), two different biopsies or even two different sections from the same biopsy may bear a variable spectrum of detectable driver mutations [2]. For example, at least 3 distinct tumor regions are needed to detect 5 key driver mutations with a 90% level of certainty [3]. ITH has also been associated with more aggressive tumor biology and poorer patient outcomes [4]. Understanding of evolutionary processes, clinical impacts, and clinical interventions in the setting of ITH are needed, but few preclinical *in vivo* models are capable of recapitulating such heterogeneity and human cancer studies are costly and challenging.

Angiosarcoma (AS), an aggressive cancer arising from vascular endothelium in anatomic sites including skin and viscera, is an important setting for the study of ITH. AS is rare in humans, but far more common in pet dogs. Tens of thousands of canine diagnoses occur annually, comprising 45–51% of splenic cancers [5]. AS in dogs, also known as hemangiosarcoma (HSA), is a complex disease that shares clinical, histopathologic and molecular characteristics with AS [6–13]. Shared molecular features include transcriptional subtypes (angiogenic, inflammatory, and adipogenic [9–11], point mutations (*TP53*, *PIK3CA*, *PTEN*, *PIK3R1* [10, 13–16], copy number gains (*PDGFRA*, *VEGFA*, *KIT*, *KDR*), and copy number deletions (*CDKN2A*/*B*, *PTEN*) [10, 12, 15, 16] and, in the angiogenic subtype, fusions (*MYO16-PTK2*, *GABRA3-FLT1*, and *AKT3-XPNPEP1*) [14], many of which hold potential as biomarkers to guide clinical management. As a naturally occurring cancer, HSA is a setting in which to perform cross-species comparative studies to improve understanding of AS development and clinical management. AS in humans and dogs is also heterogeneous in clinical presentation, clinical course, histopathology and cellular composition. Genomic study of HSA may thereby present a unique opportunity to dissect the role of ITH in an aggressive sarcoma in which genomic biomarkers are increasingly needed for clinical management. Here, we describe the results of multi-platform genomic analysis of 51 splenic masses from 43 dogs (27 HSAs, 15 benign lesions, and 1 stromal sarcoma) including matched tumor/normal exome and targeted-panel-based next generation sequencing. These data expand understanding of HSA’s inter- and intra-tumoral genomic heterogeneity and support its study as a naturally occurring model of cancer heterogeneity.

## Materials and methods

### Sample collection and nucleic acid extraction

Samples and sequencing platforms are summarized in Table 1 and S1 Table. Three splenic masses preserved in AllProtect reagent (Qiagen) and formalin from previously diagnosed HSA were used as pilot samples for evaluation of a custom canine amplicon panel. Subsequently 43 canine patients who were diagnosed with a hemoperitoneum with a ruptured splenic mass and had not received previous chemotherapeutic treatment for hemangiosarcoma were prospectively recruited to biospecimen collection within a clinical trial at 10 Ethos Veterinary Health hospitals under informed consent as previously reported [17]. This study was launched following approval from an Animal Care and Use Committee convened by Animal Clinical Investigation (Chevy Chase, MD). Briefly, whole blood and samples from splenic masses were collected at time of splenectomy. Tumor sample collection was performed within 5 minutes of the spleen being removed. Multiple 1 x 1 x 1 cm biopsies were taken from three regions of the spleen (primary tumor, tumor periphery, and normal spleen). The primary tumor section was further divided into additional geographically distinct sections for histology and sequencing where each subsequent section was placed in the following: 10% formalin for histopathology, AllProtect reagent for nucleic acid extraction, cryovials containing fetal bovine serum and 10% DMSO for cryogenic cell storage, and L15 tissue collection media for future cell line development. The assessment of diagnosis and tumor content was conducted by a single board-certified veterinary cancer pathologist (EJE). This assessment was conducted blinded to the histological diagnosis. Tumor content assessment was performed using a qualitative assessment of tumor versus normal tissue contributions to the entire submitted sample (percent of presumed neoplastic cells compared to other cell types) from hematoxylin and eosin-stained slides. The samples in formalin, AllProtect reagent, and L15 media were refrigerated at 4°C and cryovials were later frozen at -20°C until submission.

**Table 1:** Samples and sequencing platforms.

Patient Diagnosis	Total Patients	Total Samples	Samples per Sequencing Platforms
Hemangiosarcoma	27	35*	24 T/N CCAP, 3 T CCAP, 12 T/N exome
Benign Complex Nodular Hyperplasia	11	11	3 T/N CCAP
Complex Hyperplasia w/Hematoma	2	2	2 T/N CCAP
Hematoma	1	1	1 T/N CCAP
Myelolipoma	1	1	1 T/N CCAP
Stromal sarcoma	1	1	1 T/N CCAP
**Total**	**43**	**51**	**33 T/N CCAP, 3 T CCAP, 12 T/N exome**

*Pt 3, 11, 16, 22: All 3 tumor sections exome sequenced. Pt 3: All 3 tumor sections panel sequenced. Canine patients with benign or malignant splenic mass (T) and matching normal (N) when available were sequenced on with either the Canine Cancer Amplicon Panel (CCAP) or whole exome sequenced (exome). All maligant samples were confirmed to be hemangiosarcoma except for one, a stromal sarcoma.

Buffy coat and plasma were collected and stored in -20°C until nucleic acid isolation. Genomic DNA from 200uL buffy coat was isolated with DNeasy Blood and Tissue kit (Qiagen) according to manufacturer’s protocol. Tumor nucleic acid extractions were performed using the Allprep DNA/RNA/miRNA Universal kit (Qiagen) according to manufacturer’s protocol. Briefly, tumor tissue in Allprotect was first rinsed with sterile 1X phosphate buffered saline and minced with sterile scalpel. Thirty mg of the minced tissue was then homogenized using the Bullet Blender Bead lysis kit (NextAdvance) and the resulting supernatant was further homogenized with QiaShredder (Qiagen). The flow through was used for nucleic acid extraction. Quality and quantity of blood and tumor genomic DNA was performed using the Qubit Fluorometer 2.0 (ThermoFisher Scientific) and TapeStation genomic DNA assay (Agilent Technologies). Genomic DNA was stored in -20°C until sequencing library construction. RNA was extracted and stored in -80°C for future use as per manufacturer’s instructions.

### Targeted amplicon sequencing and analysis

Targeted amplicon sequencing was performed on genomic DNA from matched blood and DNA from individual patient AllProtect tumor sections with the highest tumor content based on evaluation of adjacent FFPE. A custom canine HSA cancer amplicon sequencing panel consisting of 330 amplicons targeting exons and hotspot regions in 68 genes, with amplicon sizes ranging from 91–272 bp was developed (S2 Table). Primers were pooled in two multiplexed pools to separate adjacent amplicons and any amplicons with high potential for cross-amplification using *in silico* PCR. Sequencing libraries were constructed using droplet-based PCR amplification following the manufacturer’s protocols for the ThunderBolts Cancer Panel with specific modifications (RainDance Technologies) as previously described [18]. Paired-end sequencing was performed on an Illumina MiSeq generating 275bp reads. Sequencing metrics are shown in S3 Table. Analysis tools and parameters are shown in S4 Table. Sequencing reads were demultiplexed and extracted using Picard 2.10.3 [19]. Sequencing adapters were trimmed using ExpressionAnalysis ea-utils [20] and quality of fastq files were assessed with FastQC v0.11.5 [21]. Sequencing reads were aligned to CanFam3.1.75 using BWA-MEM v0.7.8 [22]. Custom in-house scripts based on SAMtools v1.2 [23] were used to create pileups for every sample. Pileups were analyzed in R to call SNVs and indels. For each potential non-reference allele at each targeted locus in a sample, we evaluated the distribution of background noise across all other sequenced samples. To call a variant, we required the observed non-reference allele is an outlier from the background distribution with a Z-score ≥ 5. The z-score is calculated by subtracting the variant allele fraction from the population mean, then dividing by the population standard deviation. In addition, we required tumor depth ≥100x, variant allele fraction ≥10%, number of reads supporting the variation ≥10, and allele frequency in the germline sample <1%. We utilized stringent cutoffs for amplicon sequencing in order to minimize false positives due to PCR artifacts and duplications. Finally, variant calls were manually curated by visualization in IGV v2.4.9. All sequencing data have been deposited in the Sequencing Read Archive under BioProject PRJNA677995.

### Exome sequencing and analysis

Genomic DNA from blood and all 3 tumor sections (AllProtect samples) from 4 HSA patients (patients 03, 11, 16, 22) underwent whole exome sequencing using the Agilent SureSelectXT CD Canine All Exon V2 (Agilent Technologies), a community-based design that contains 982,789 probes covering 19,459 genes [24]. The BED file is available from Agilent Technologies by request. Exome libraries were sequenced on the Illumina NovaSeq 6000 producing paired end reads of 100bp. Analysis tools and their parameters are shown in S4 Table. FASTQ files were aligned to the canine genome (CanFam3.1.75) using BWA v0.7.8 [22]. Aligned BAM files were realigned and recalibrated using GATK v3.3.0 [25] and duplicate pairs were marked with Picard v1.128 [19]. Somatic single nucleotide variants (SNV) were identified only when called by two or more of the following callers: Seurat v2.6 [26], Strelka v1.0.13 [27] and MuTect v1.1.4 [28] and with an allele fraction of > 5%. Whereas a 10% variant allele fraction filter was applied to CCAP, the exome somatic cutoff was more permissive at 5% due to differences in platform performance. This filtering strategy was applied to interpretation of the variants as candidate driver mutations and to tumor mutation burden analyses. Variants called by CCAP and WES are described in S5 and S6 Tables with their associated variant allele fractions (AFs). The majority of the candidate mutations identified via CCAP that were below the 10% threshold occurred at a variant AF of 1–2% and were not likely pathogenic driver mutations. Germline SNVs were called using Haplotype Caller (GATK v3.3.0) [29], Freebayes [30] and samtools-Mpileup [31]. Only those with allele fraction > 2% were included. Variant annotation was performed with SnpEff v4.3 [32]. TMB was calculated as the total number of somatic mutations per haploid callable Megabase (Mb) from WES [33]. Percent tumor content was inferred from somatic mutation data utilizing Sequenza [34]. The exome data corresponding to normal blood and tumor sections are uploaded to Sequencing Read Archive under BioProject PRJNA677995.

## Results and discussion

Based on the recognized clinical, histologic, and cellular heterogeneity of canine HSA we sought to characterize its degree of inter- and intra-tumoral heterogeneity through a prospective multicenter study [17] in which genomic analysis was performed on untreated benign and malignant tissues from 43 pet dogs presenting to emergency hospitals with hemoperitoneum after splenic mass rupture. Samples and sequencing platforms are summarized in Table 1 and S1 Table. Of 43 cases, 27 (63%) were diagnosed with HSA, 15 (34%) were diagnosed with benign lesions (11 benign complex nodular hyperplasia, 2 complex hyperplasia with hematoma, 1 hematoma, and 1 myelolipoma) and 1 dog was diagnosed with stromal sarcoma. Tumors were collected from three geographically distinct splenic regions. Each of 3 sections was sub-divided for histopathology or genomics. Tumor content estimated via histopathology in three geographically distinct primary tumor regions from 23/27 HSA patients ranged from 0 to > 95% (median 10%, S1 Table). The range of tumor cell content and the high frequency of tumor sub-sections without identifiable tumor cells in sections that were grossly suspected to consist of primary tumor both underscore the degree of histologic and cellular heterogeneity in HSA and potential challenges associated with genomic discovery and diagnostic studies in this tumor type. Images of stained tumor sections adjacent to samples utilized in this study are shown in S1 Fig that are representative of this diversity.

**Fig 1:**
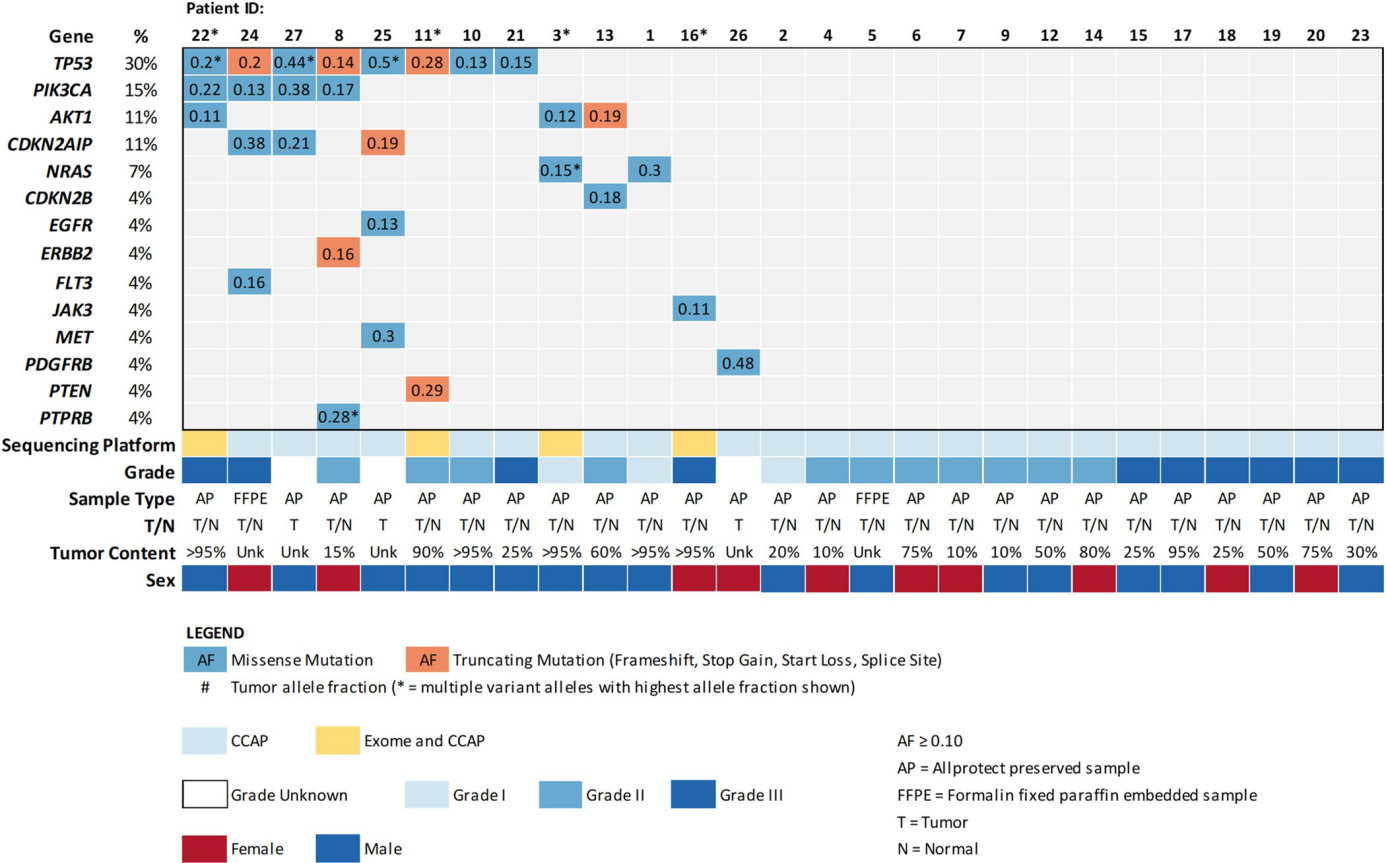
The landscape of somatic SNVs in splenic hemangiosarcoma.

To identify somatic single nucleotide variants (SNVs), we utilized a custom canine cancer next generation sequencing amplicon panel [35] with regions covering commonly mutated genes in AS and HSA (S2 Table) [7, 12, 16] including 330 exonic regions of canine orthologs of 67 commonly mutated human cancer genes. The section with the highest tumor content out of the three sections was sequenced. Matched normal, when available, and tumor tissue was sequenced to an average depth of 1,910x and 1,670x, respectively (S3 Table). We identified 34 putative somatic SNVs across this cohort at an allele frequency (AF) ≥ 10% (S5 Table). At least one somatic mutation with an AF ≥ 10% was seen in 13/27 (48%) of the sequenced tumors (Fig 1). The median AF for these 34 SNVs was 0.22 (range 0.10–0.50). *TP53* was the most frequently mutated gene, with 11 mutations occurring across 8 patients (30%). Of 11 *TP53* mutations, 8 were missense with most occurring in human-equivalent pathogenic hotspots in the DNA-binding domain. Single cases of splice acceptor (c.530-2A>C), stop gain (R296*), and frameshift (T243fs) mutations were identified. *PIK3CA* mutations were identified in 4 patients (15%). *PIK3CA* H1047L was the only recurrent point mutation, identified in 2 cases. The third and fourth cases bore H1047R and G1049R mutations. Amino acid 1047 is the most frequently mutated *PIK3CA* hotspot in human cancers, previously shown to be mutated in HSA (30–46% of cases) [13, 16, 36]. *AKT1* was mutated in 3 cases (11%) with two potentially pathogenic missense mutations, G37D and R23W, and one frameshift, L52fs, with unknown significance. Additional likely pathogenic mutations in single patients included *CDKN2B* R105Q and *NRAS* Q61R. Variants of unknown significance (VUS) include *CDKN2AIP* Q67R, a gene deleted in HSA via aCGH studies [12], an *ERBB2* splice region variant, *FLT3* N703S, *JAK3* M724T, *PTEN* H39fs, and *PTPRB* L1284P and S1965P. Of the 7 panel-sequenced benign cases, only Patient 37 (benign hyperplasia) bore a somatic SNV in a cancer gene—a VUS impacting *PTPRB*. Patient 43 (stromal sarcoma) bore a likely pathogenic missense mutation at R789C in *GNAS*. These driver mutations and their frequencies resemble those identified in HSA in other studies [13, 16, 37]. The genomic landscape of HSA confirmed here provides a framework to guide study of HSA development while also guiding therapeutic strategies under a precision medicine paradigm in which drug-biomarker relationships may exist in HSA (S2 Fig).

Recurrent, potentially pathogenic mutations were identified in 24 matched normal/tumor and 3 tumor-only FFPE- or AllProtect-preserved splenic hemangiosarcomas. As indicated by colored boxes, missense and truncating mutations identified by amplicon panel-based sequencing (CCAP) with allele frequencies ≥ 0.1 are shown. Allele frequencies are indicated in colored boxes. Tumor content percentage and tumor grade are shown where available. Patient IDs with asterisks indicate samples that were also exome-sequenced.

Intratumoral genomic heterogeneity presents challenges to cancer precision medicine (i.e. the utilization of genomic diagnostics to guide the clinical management of cancer patients). For example, variability in subclonal frequency of driver mutations may modulate treatment response. Additionally, such variability means that any given biopsy utilized for genomic analysis may not contain driver mutations with predictive associations even if these mutations are highly abundant in major lineages in the tumor. This study has confirmed the presence of genomic variants and intratumoral heterogeneity in canine HSA that can facilitate hypothesis testing and methods development to meet these needs. For example, *NRAS* and *PIK3CA* mutations such as those described in HSA have been associated with *MEK* and *PIK3CA* inhibitor responses, respectively, in human cancers and may also be associated with such responses in canine cancers. Such responses, however, may also be dependent on intratumoral heterogeneity and will certainly be dependent on the ability to detect subclonal or low allele-frequency mutations. Basket and umbrella clinical trials that test such hypotheses in canine HSA will hold value broadly for understanding heterogeneous human cancers and will be especially valuable in less common human cancers such as angiosarcoma.

In order to more deeply explore heterogeneity, we next directly measured ITH via whole exome sequencing (WES) of three geographically distinct splenic sections from 4 patients (Patients 3, 11, 16, 22) along with their matching constitutional DNA from peripheral blood, achieving average sequencing coverage of 214x for tumors and 211x for normals. Patients with known pathogenic driver mutations according to panel-based sequencing and those with high tumor content according to pathology for all 3 sections were selected (S1 Table). Based on WES, these tumors overall bore a low somatic tumor mutation burden (TMB), median = 0.7 mutations/Mb (range: 0.3–2.9 mutations/Mb, Table 2). Tumor content estimates, determined informatically by Sequins’ using AFs from exome sequencing data, suggested a median of 21% (range 10–57%, Table 2) whereas pathology estimates suggested a median of 75% (range 25%->95%, S1 Table). Importantly, although all sequencing was performed on AllProtect samples immediately adjacent to FFPE sections evaluated for tumor content by pathology, it is still possible that tumor content of the AllProtect sections did not directly reflect that of the FFPE. Nonetheless, discordance between genomics-based and pathology-based tumor content estimates is well-recognized in the field and is likely further influenced here by low TMB, physiologic heterogeneity, and genomic heterogeneity in these cases.

**Table 2:** Tumor mutation burden and tumor content of exome-sequenced samples.

Sample	Tumor Section	TMB (Mutations/Mb)	Histologic Tumor Content (%)	Bioinformatic Tumor Content (%)
Pt 3	1	0.315	60%	10%
Pt 3	2	0.265	75%	10%
Pt 3	3	1.106	95%	10%
Pt 11	1	2.855	45%	30%
Pt 11	2	2.582	50%	52%
Pt 11	3	2.620	90%	43%
Pt 16	1	0.702	>95%	40%
Pt 16	2	0.682	>95%	57%
Pt 16	3	0.047	50%	10%
Pt 22	1	0.246	25%	12%
Pt 22	2	1.028	>95%	40%
Pt 22	3	0.033	75%	10%

**Fig 2:**
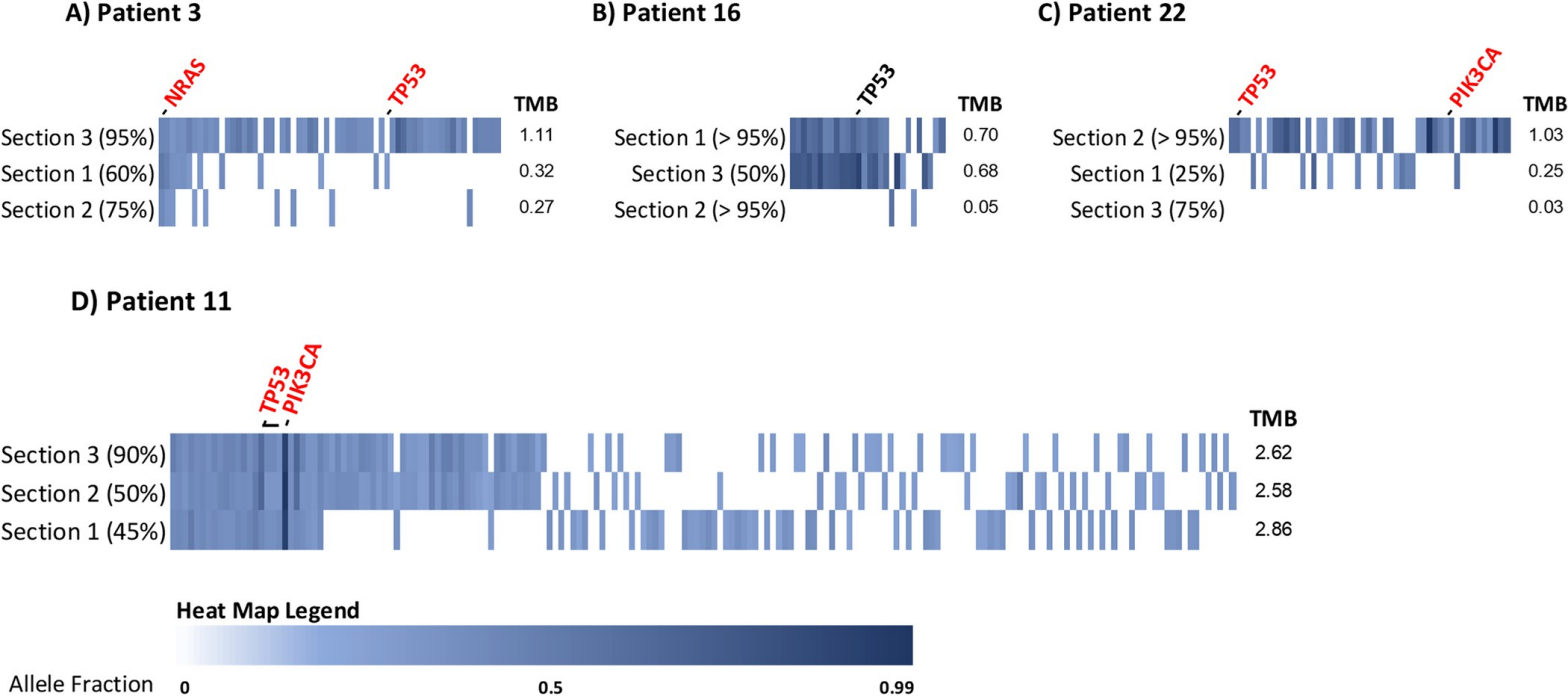
Intratumoral genomic heterogeneity in splenic hemangiosarcoma.

Analysis of intersecting somatic coding mutations across 3 tumor regions from 4 patients by WES identified substantial intratumoral genomic heterogeneity (Fig 2A–2D, S6 Table). Between 0 and 64% of SNVs were detected in at least 2 regions and 0–13% were detected in all 3 regions across these 4 patients. In Patient 3, 9/62 (15%) SNVs were shared in 2 or more regions and 3 (5%) were shared in all regions (Fig 2A). The hotspot cancer driver, *NRAS* Q61R, was detected in all regions, although the hotspot *TP53* Y210C (equivalent to human pathogenic *TP53* Y220C mutation) was only detected in a single section. In Patient 16, 18/28 (64%) SNVs were shared in regions 2 and 3, including a missense mutation of unknown pathogenicity *TP53* Y152S (equivalent to human *TP53* Y163S). No mutations were shared by all regions or between regions 1 and 3 (Fig 2B). For Patient 22, no mutations were shared among all regions (Fig 2C). Only region 2 bore candidate driver missense mutations (also identified by CCAP): *TP53* G256E and R272H (likely pathogenic mutations equivalent to human *TP53* G266E and R282H) and *PIK3CA* H1047L. In Patient 11, 61/181 (34%) SNVs occurred in 2 or more regions and 24 (13%) were shared among all regions (Fig 2D). Two driver mutations, *TP53* T243fs and *PIK3CA* G1007R, were present in all regions. In most cases where a tumor section was sequenced by both CCAP and WES, mutation concordance between CCAP-targeted and WES-targeted genomic regions was seen with the exception of *PIK3CA* G1007R in Patient 11 (detected by WES, but not CCAP likely due to its occurrence in a CCAP primer region) and *AKT1* G37D in Patient 22 (detected in CCAP but not WES likely due to the increased sensitivity of CCAP sequencing). Overall, of 8 total known pathogenic or likely pathogenic cancer driver mutations (based on inference from human-aligned mutation positions) detected in these patients, half were shared among all distinct tumor regions from the same dog whereas the other half were present in only a single section. This variability in somatic mutation frequency across different geographic regions from the same tumor supports that significant genomic heterogeneity exists in HSA. Alongside cellular/physiologic heterogeneity (i.e. tumor content variability), this genomic heterogeneity holds implications for understanding HSA development and diagnosis while positioning HSA as a model system to understand and manage ITH.

Intratumoral heterogeneity was assessed via whole exome sequencing of 3 distinct geographic regions from 4 canine HSA patients selected based on high average tumor content across multiple samples as well as presence of one more known or likely driver mutations from CCAP-based sequencing of the highest tumor content section. Individual patient oncoprints show coding, nonsynonymous mutations within each tumor section. Shared genomic events align vertically between the various tumor regions within a given patient. Each section is also annotated with tumor content assessment based on pathologic review in parentheses. All known pathogenic or likely pathogenic driver mutations (based on translated protein alignment and inference from human ClinVar annotation) are annotated and indicated in red text. TMB = tumor mutation burden.

Phenotypic and genomic heterogeneity are well-established and increasingly well-understood features of human solid tumors that hold relevance for understanding cancer’s genesis, its development, and its varied clinical behaviors. They also have bearing on cancer diagnosis via traditional diagnostics, like histopathology, and novel diagnostics such as genomic tumor profiling. Here we describe genomic heterogeneity in a common naturally occurring canine cancer, hemangiosarcoma (HSA), that has long been recognized as a clinically, physiologically, and histologically complex cancer. Through multi-platform (exome and gene panel) genomic analysis of 28 HSAs focused on identification of somatic SNVs, we identified marked inter- and intra-tumoral heterogeneity in canine HSA. First, we identified candidate somatic cancer driver mutations in 50% of cases. Notably, the majority of the 258 somatic SNVs detected in this cohort occurred at low tumor AFs < 10% (median of 3% for all SNVs), though all but one of the tumors bearing a known somatic driver contained at least one somatic mutation with an AF ≥ 10% (Fig 1). This observation is related at least in part to the overall low tumor cellularity and extensive physiologic heterogeneity of HSA cases, a feature of this cancer type that presents challenges for both traditional and genomic diagnostics. Although we conducted carefully controlled and extensive pathologic review of tumor content from tissue adjacent to sequenced tumor tissue in order to prioritize tumor sections with high neoplastic cellularity, tumor content was still ~55% according to pathology review on average where available. The low median AF of SNVs, despite sequencing of tumor tissue with relatively high estimated tumor cellularity, also supports the existence of subclonal heterogeneity in hemangiosarcoma.

These data also contribute to our growing understanding of HSA’s genomic underpinnings. The genomic landscape in this cohort was similar to what has been previously described. *TP53* was the most commonly mutated gene (30%) followed by *PIK3CA* (15%), *AKT1* (11%), and *CDKN2AIP* (11%), though frequencies of some detected mutations differ. Cross-study variation in this landscape, such as the lower rate of *PIK3CA* mutations identified in our cohort relative to others (15% versus 30–46%), likely reflects natural variation by HSA anatomic site, clinical characteristics, and breed in addition to variation in sequencing platforms and analysis approach. It underscores the need for expanded genomic study in very large cohorts. It also remains possible that other drivers are present in this cohort in regions not included in the panel in addition to copy number variation, translocations, and fusions. Large, integrated genomic studies that incorporate multiple sequencing platforms and mutation identification types across a diversity of breeds and anatomic sites will ultimately be necessary for refining our view of HSA’s genomic landscape.

Finally, we identified significant intratumoral genomic regional heterogeneity via analysis of multiple sections from the same tumor. In two cases, known pathogenic driver mutations such as those occurring in *NRAS*, *TP53*, and *PIK3CA* were detectable in all sections from the same tumor at AFs ranging from 10–84%, supporting that in some cases, truncal driver mutations arise in clonal fashion and are universally present in the tumor cells (Fig 2). However, two cases also contained known pathogenic driver mutations that were only detected in a single section including *TP53* Y210C, G256E, and R272H as well as *PIK3CA* H1047L at AFs of 5%, 19%, 19%, and 9%, respectively. These drivers were all detected in sections with the highest tumor content based on pathologic assessment, but were notably not detected in the lower tumor content sections (90% versus 60% and 75% for Patient 3 and >95% versus 25% and 75% for Patient 22). Also notable is that the cases with lower regional concordance contained at least one section with low TMB and low bioinformatically inferred tumor content even though histological review of tumor content may have been high (Table 2 and Fig 2). Conversely, Patient 11 bore complete concordance for known driver mutations across regions and additionally bore the consistently highest TMB in these sections despite lower histologic tumor content assessments for two regions.

Concordance between histologic and bioinformatic tumor content estimates was low (S3 Fig). We also do not observe a significant relationship between either bioinformatic or histopathologic assessment of tumor content and tumor mutation burden or median variant AF in this small sample set, although bioinformatic tumor content assessment more closely correlates with TMB and median AF as we would expect given that these methods are inter-dependent. Thus, overall these analyses support that the observed variation in tumor content is not sufficient to explain the more pronounced genomic heterogeneity we observed based on sequencing results. However, both because we did not perform macro- or micro-dissection in order to enrich for tumor cells and also because we sequenced AllProtect sections adjacent but not the same as FFPE sections reviewed by pathology, it remains possible that cellular heterogeneity is a confounding factor and a significant source of the variability observed between tumor sections. Future studies comparing gross and macro- or microdissected tumor sections will be critical for further expanding a detailed view of the degree of ITH in HSA. Nonetheless, our data strongly support that HSA follows a model of branched evolution with truncal driver mutations, but significant branching evolution characterized by heterogeneous mutations that are likely predominantly passengers. They also support the importance of accounting for cellular heterogeneity in HSA sampling for genomic characterization and more broadly for diagnostics.

## Conclusions

Overall, our multi-platform genomic analysis of 52 splenic tumor samples confirms the presence of key driver mutations in HSA while also revealing extensive ITH. These data provide new perspective on the low or variable tumor cellularity, high physiologic heterogeneity, and the possibility of significant subclonal heterogeneity in HSA, thereby highlighting the importance of accounting for these features in future genomic research and for diagnostics. We can also now begin to leverage our understanding of the presence of this genomic heterogeneity in these naturally occurring cancer models to develop and adapt strategies to translate the value of genomic medicine to human and canine cancer patients.

## Supporting information

S1 Table Extended demographic and clinical annotation. (XLSX)

S2 Table Genes included in the Canine Cancer Amplicon Panel (CCAP). (XLSX)

S3 Table CCAP and exome sequencing statistics. (XLSX)

S4 Table Informatic tools, versions, and parameters utilized in analysis of Canine Splenic Lesions via Exome and CCAP sequencing. (XLSX)

S5 Table Somatic SNVs and indels identified by CCAP sequencing in Canine Splenic Lesions. (XLSX)

S6 Table Somatic SNVs and indels identified by exome sequencing in HSA. (XLSX)

S1 Fig Histological heterogeneity in canine hemangiosarcoma. Representative images of hematoxylin and eosin-stained canine hemangiosarcoma sections adjacent to tumors sequenced in this study. 100x magnification with 50 μm scale bars shown on the lower left. (A) Patient 3. This tumor has low cell density with large cavernous blood-filled sinusoidal structures lined by monotonous endothelioid cells and separated by thin trabeculae. (B) Patient 12. This tumor has variable density with solid areas of tumor cells and disorganized collapsed blood-filled clefts as well as areas with large cavernous blood-filled sinusoidal structures. The atypical endothelioid cells have plump nuclei with anisokaryosis.
(TIF)

S2 Fig Models for precision medicine clinical trials in naturally occurring Canine Hemangiosarcoma. (TIF)

S3 Fig Evaluation of correlation between histopathologic and bioinformatic assessment of tumor content and tumor mutation burden. For each of the cases with three independently sequenced tumor sections, the Pearson correlation coefficient was calculated for: A) Median variant allele fraction (AF) versus histologically and bioinformatically determined tumor content estimates; and B) Tumor mutation burden (TMB) versus histologically and bioinformatically determined tumor content estimates.
(TIF)
