# Supplementary figures and images for: Genomic landscapes of canine splenic angiosarcoma (hemangiosarcoma) contain extensive heterogeneity within and between patients
Source: PLoS One. 2022 Jul 22;17(7):e0264986. doi: 10.1371/journal.pone.0264986 (PMC9307279; doi:10.1371/journal.pone.0264986)

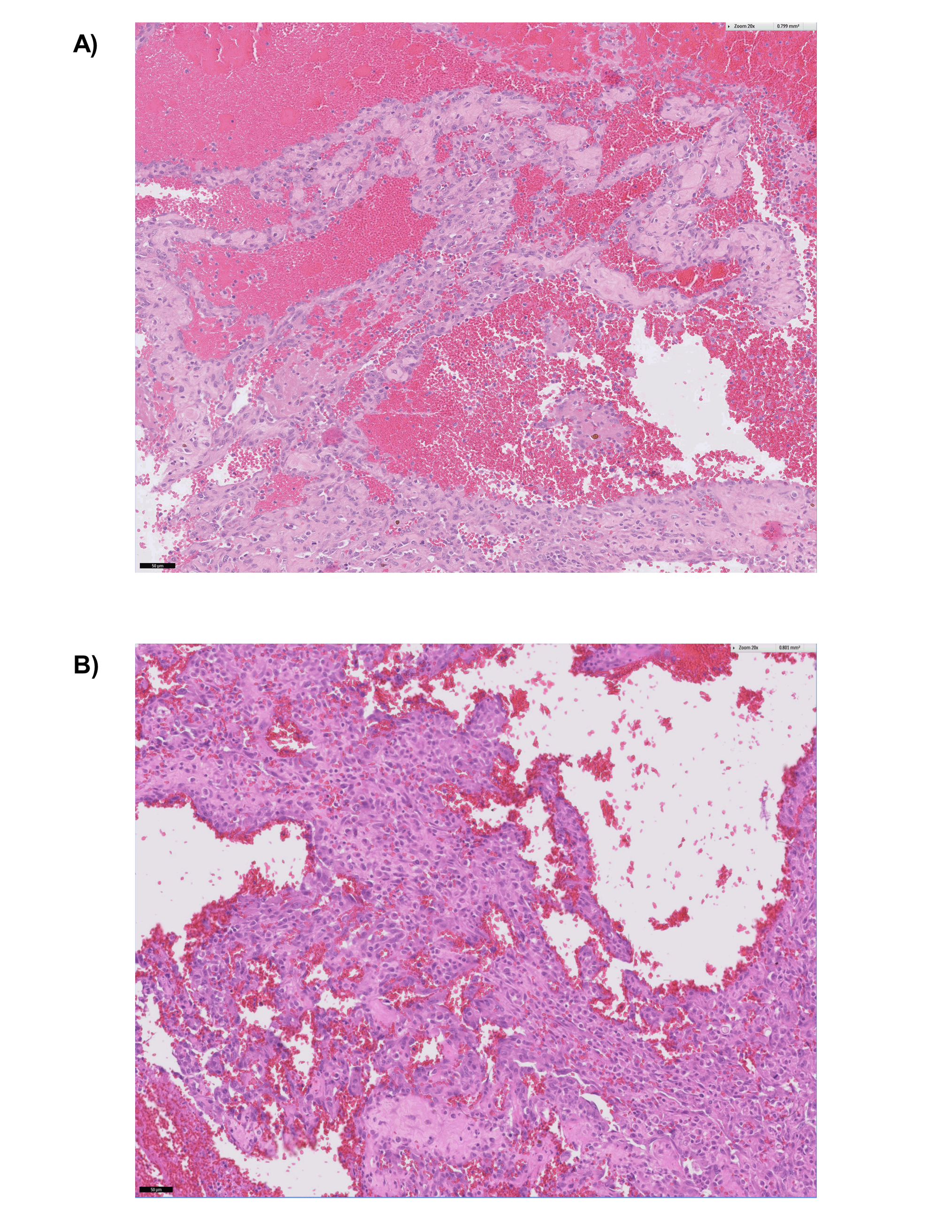

Supplement: S1 Fig — Representative images of hematoxylin and eosin-stained canine hemangiosarcoma sections adjacent to tumors sequenced in this study. 100x magnification with 50 μm scale bars shown on the lower left. (A) Patient 3. This tumor has low cell density with large cavernous blood-filled sinusoidal structures lined by monotonous endothelioid cells and separated by thin trabeculae. (B) Patient 12. This tumor has variable density with solid areas of tumor cells and disorganized collapsed blood-filled clefts as well as areas with large cavernous blood-filled sinusoidal structures. The atypical endothelioid cells have plump nuclei with anisokaryosis. (TIF) [file pone.0264986.s007.tif]

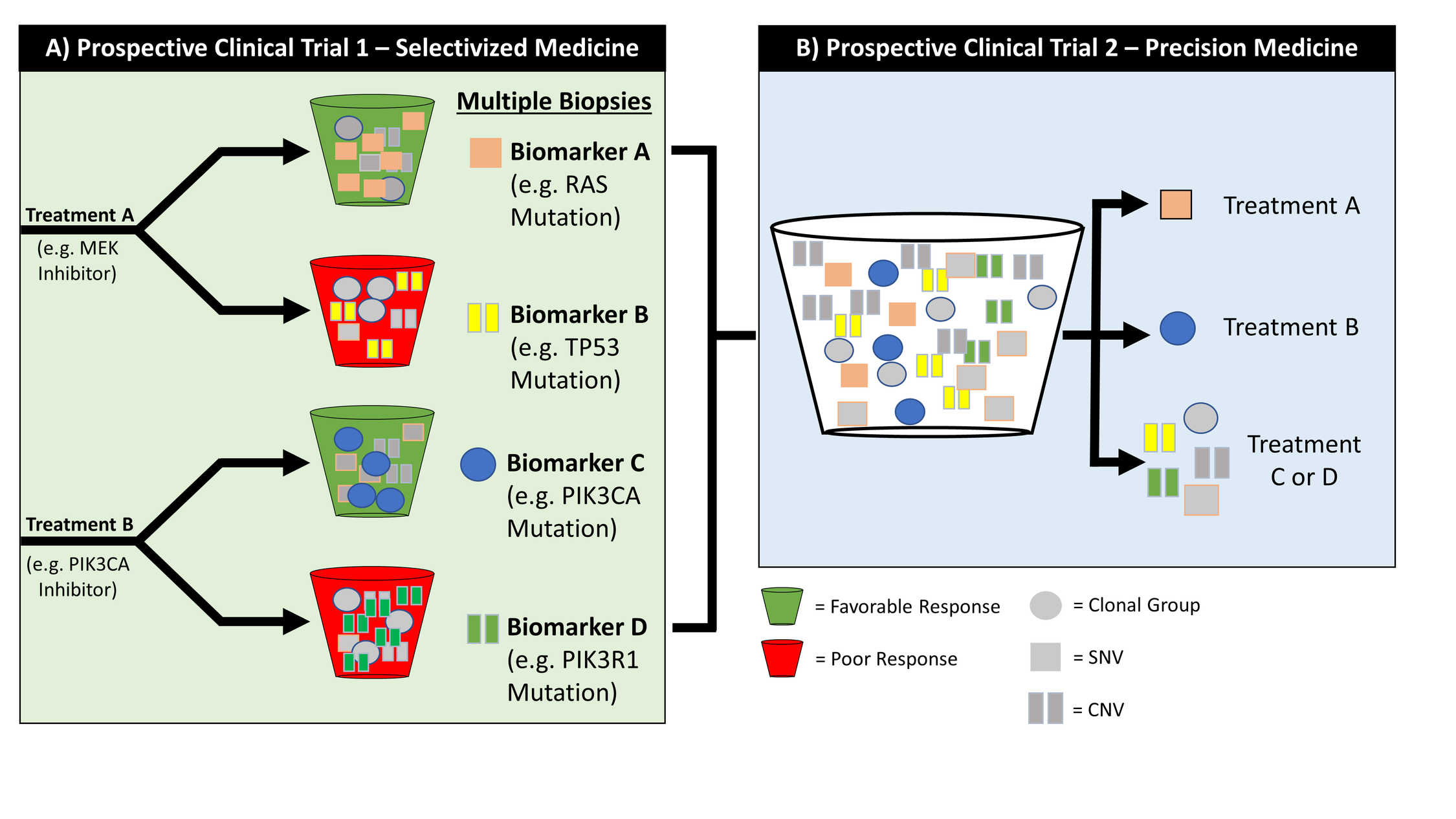

Supplement: S2 Fig — (TIF) [file pone.0264986.s008.tif]

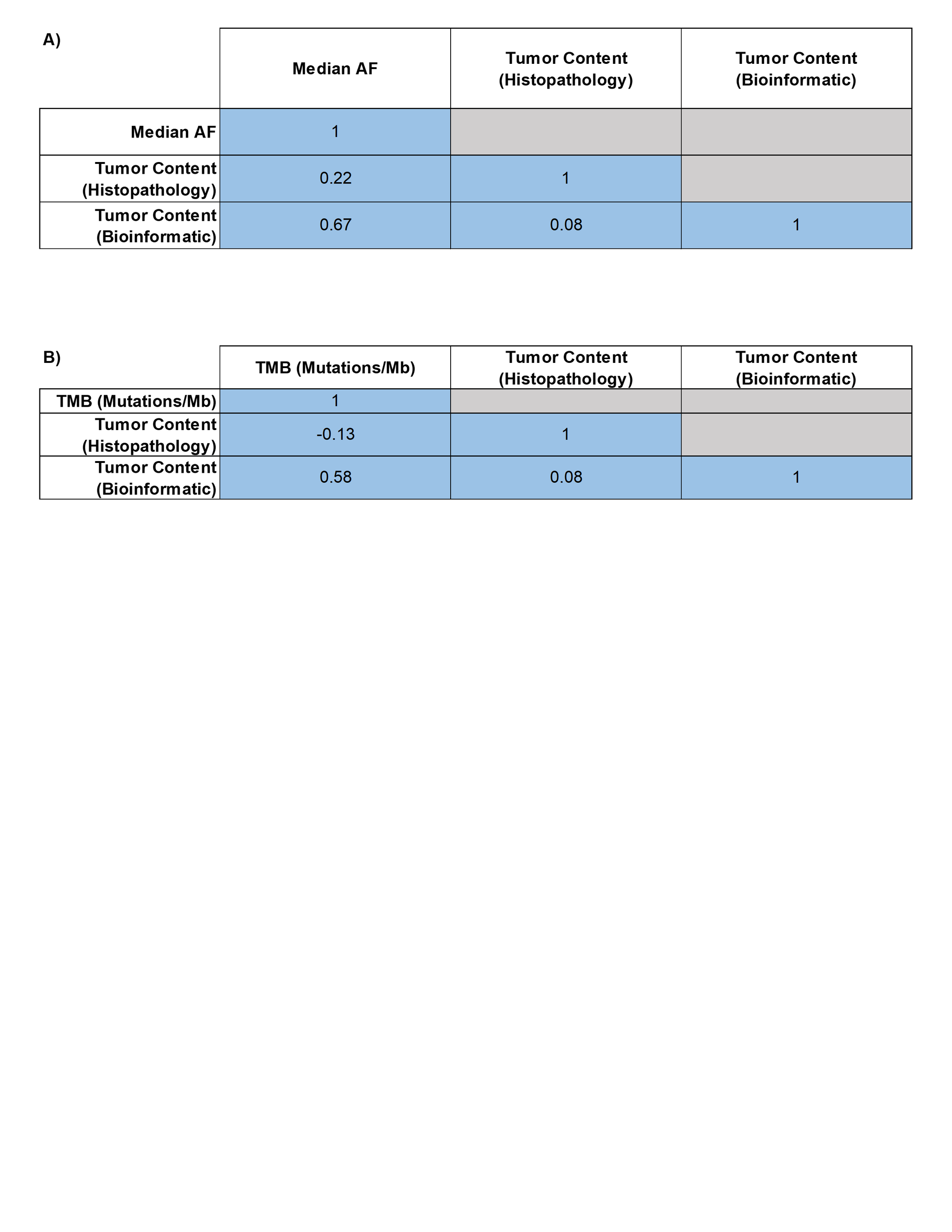

Supplement: S3 Fig — For each of the cases with three independently sequenced tumor sections, the Pearson correlation coefficient was calculated for: A) Median variant allele fraction (AF) versus histologically and bioinformatically determined tumor content estimates; and B) Tumor mutation burden (TMB) versus histologically and bioinformatically determined tumor content estimates. (TIF) [file pone.0264986.s009.tif]
